# SOX4 promotes high-glucose-induced inflammation and angiogenesis of retinal endothelial cells by activating NF-κB signaling pathway

**DOI:** 10.1515/biol-2022-0045

**Published:** 2022-04-25

**Authors:** Haifeng Wei, Quan Gu

**Affiliations:** Department of Ophthalmology, Tongxiang First People’s Hospital, No. 1918 Jiaochang East Road, Jiaxing, Zhejiang Province, 314500, China

**Keywords:** diabetic retinopathy, SOX4, migration, angiogenesis, NF-κB pathway

## Abstract

Diabetic retinopathy (DR) is a type of main microvascular complication of diabetes mellitus (DM) and an important factor that causes blindness in adults. SOX4 is a transcription factor expressed in the pancreas and is essential for normal endocrine pancreatic development. However, the effect and the regulatory mechanism of SOX4 on DR have not been reported. In the present study, upregulation of SOX4 was found in DM patients, particularly in DR patients and mice models. The *in vitro* experiments showed that SOX4 depletion increased the viability and inhibited the inflammation level of human retinal endothelial cells (HRCECs) induced by high glucose. Besides, SOX4 knockdown inhibited the migration and angiogenesis of HRCECs upon high glucose treatment. Mechanically, depletion of SOX4 inhibited the NF-κB pathway. Therefore, SOX4 could serve as a promising target for DR treatment.

## 1 Introduction

Diabetic retinopathy (DR) is the main microvascular complication of diabetes mellitus (DM) and is a leading factor that causes blindness [1,2]. The main pathological changes of DR include retinal cell apoptosis and angiogenesis [3]. Previous studies demonstrated that the progression of DR was accompanied by oxidative stress, mitochondrial dysfunction, and inflammation [2,4,5,6]. Because the highly complex pathogenesis of DR has not been fully elucidated, it is thus necessary to study and search for new therapeutic targets. In particular, inflammation and angiogenesis are two critical factors affecting DR progression.

The SOX family on the Y chromosome is an important set of transcriptional regulators involved in many developmental processes, including the endocrine pancreas development [7,8]. SOX4 is a transcription factor expressed in the pancreas and is essential for normal endocrine pancreatic development [9,10]. Studies have found that overexpression of SOX4 regulates the CXCL12 promoter in liver cancer cells to enhance tumor-induced angiogenesis, which contributes to distant tumor metastasis and causes poor prognosis in liver cancer patients [11]. Other studies have reported that SOX4 can be upregulated explicitly by retinoic acid and interleukin (IL)-1 treatment, a key transcription factor related to the pathogenesis of osteoarthritis [12]. In addition, increased SOX4 expression inhibits insulin secretion by upregulation of STXBP6 and increases the risk of diabetes [13]. The role of SOX4 in metabolism and immunity has also been further studied. For example, SOX4 represses host innate immunity to facilitate pathogen infection by hijacking the Toll-like receptor (TLR) signaling networks [14]. However, its effects on DR have not been reported.

Inflammation is the main pathological feature of DR, and inhibition of inflammation has been found to significantly alleviate the development of DR [15]. The transcription factor NF-κB is responsible for expressing a variety of pro-inflammatory cytokines and plays an important role in causing inflammation in many diseases [16]. It has been reported that Erianin could reduce microglia-induced retinal inflammation and DR by inhibiting the hyperglycemia-mediated ERK1/ERK 2-NF-κB axis [17]. In addition, it has been reported that SOX4 can promote the migration and invasion of melanoma cells by activating the NF-κB pathway [18]. Here, this study aimed to clarify the mechanism underlying SOX4-mediated NF-κB pathway in the DR model.

A series of experiments on high-glucose-induced human retinal endothelial cell (HRCEC) injury were performed to verify it. The results showed that the depletion of SOX4 could reduce the inflammatory level and inhibit the angiogenesis of HRCECs via targeting the NF-κB pathway.

## 2 Materials and methods

### 2.1 Samples

The peripheral blood of healthy people, DR patients, and treatment groups from our hospital was collected to detect the mRNA level of SOX4. The patient has signed informed consent; 8-week-old female STZ-induced DB model mice (with and without retinopathy) were purchased from the Beijing weitonglihua company. The expression level of SOX4 protein was detected using immunoblot after the mouse eyeball was taken out.


**Ethical approval:** The research related to animal use has been complied with all the relevant national regulations and institutional policies for the care and use of animals.

### 2.2 Antibodies

SOX4 antibody (1:500 dilutions, ab243739, Abcam, Cambridge, UK), vascular endothelial growth factor (VEGF) antibody (1:1,000 dilution, ab32152, Abcam), p65 antibody (1:500 dilution, ab32536, Abcam), p-p65 antibody (1:500 dilution, ab76302, Abcam), IκBα antibody (1:1,000 dilution, #9242, Cell signaling, USA), p- IκBα antibody (1:500 dilution, #2859, Cell signaling, USA), GAPDH antibody (1:2,000 dilution, ab8245, Abcam).

### 2.3 Cell culture, high glucose treatment, and transfection

HRCECs were cultured in Dulbecco’s modified Eagle’s medium (DMEM), supplemented with 10% fetal bovine serum at 37°C in a 5% CO_2_ incubator. HRCECs were treated with 33 mM glucose for 24 h. The siRNAs were from RioBio and transfected into cells by Lipofectamine 2000 (11668019; Invitrogen, Carlsbad, CA, USA). For siRNA transfection, 10 μL of siRNAs of SOX4 was added into 200 μL of OPTI medium for 5 min; 5 μL of lipofectamine was then added into 500 μL of OPTI-MEM for 5 min, mixed for 20 min, and added into cells. After 24 h, the subsequent assays were performed.

### 2.4 Immunoblot assay

Cell samples were lysed with lysis buffer (60 mM Tris–HCl; pH, 6.8; 2% sodium dodecyl sulfate [SDS]; 20% glycerol; 0.25% bromophenol blue; 1.25% 2-mercaptoethanol; and protease inhibitor cocktail, Beyotime Institute of Biotechnology, Beijing, China). Total protein was separated using 10% SDS–polyacrylamide gel electrophoresis (PAGE) and sequentially transferred onto poly (vinylidene fluoride) (PVDF) membranes (IPSN07852; EMD Millipore Corp, Billerica, MA, USA). The PVDF membranes were then blocked with 5% dry milk at room temperature for 2 h in tris-buffered saline with Tween (TBST) and subsequently incubated with the primary antibodies in TBST buffer for 1.5 h. Finally, the membranes were maintained with horseradish-peroxidase-conjugated antibodies for another 1 h. Signals were then visualized using the enhanced chemiluminescence (ECL) kit.

### 2.5 Quantitative PCR (qPCR) assays

Trizol (15596026; Invitrogen, Carlsbad, CA, USA) served as an isolation kit to extract RNAs from cells. Then, the RNAs were reverse-transcribed using M-MLV reverse transcriptase (Promega, Madison, WI, USA). Total mRNA was then reversely transcribed using a cDNA synthesis system. Quantitative polymerase chain reaction (qPCR) was performed using an SYBR Ex Taq kit (Takara, Japan). The following thermocycling conditions were used: initial denaturation at 95°C for 3 min, followed by 30 cycles of denaturation at 95°C for 30 s, annealing at 58°C for 30 s, and extension at 72°C for 30 s. The 2^−ΔΔCq^ method was used to quantify the results. Subsequently, the mRNA level was normalized to GAPDH. The primer sequences were as follows: tumor necrosis factor (TNF)-α: 5′-ATGAGCACTGAAAGCATGATC-3′ and 5′-TCACAGGGCAATGATCCCAAAGTAGACCTGCCC-3′, IL-6: 5′-ATGAACTCCTTCTCCACAAGC-3′ and 5′-CTACATTTGCCGAAGAGCCCTCAGGCTGGACTG-3′, IL-8: 5′-ATGACTTCCAAGCTGGCCGTG-3′ and 5′-TTATGAATTCTCAGCCCTCTTCAAAAACTTCTC-3′.

### 2.6 3-(4,5-Dimethylthiazol-2-yl)-2,5-diphenyltetrazolium bromide (MTT) assay

After transfection for 48 h, HRCECs were maintained for 48 h and then treated with MTT (Beyotime Institute of Biotechnology, Beijing, China) for 3 h and washed with phosphate-buffered saline (PBS). Cells were then extracted using 150 μL of dimethyl sulfoxide, and the absorbance value at a wavelength of 570 nm was measured and finally analyzed using a Multifunctional enzyme marker (Thermo Fisher, San Jose, CA, USA).

### 2.7 Enzyme-linked immunosorbent assay (ELISA) assay

HRCECs were lysed with PBS and minced with magnetic beads, and lysates were collected and centrifuged. Concentrations of TNF-α, IL-6, and IL-8 in HRCECs were measured using ELISA kit (R&D Systems, Minneapolis, MN, USA). In brief, the sample was added to the reaction well (100 µL per well). At the end of incubation, the reagent was poured out of the reaction well, added 250 µL of 1× PBST buffer, then quickly poured it out, and repeated it twice. Next, an enzyme-labeled antibody was added, incubated for 2 h at 100 µL per well, and finally washed on the plate.

### 2.8 Transwell assay

Cells were resuspended in serum-free medium and were plated into the upper chambers to induce the migration toward the place containing the complete medium. After 24 h, cells in the top were removed, and the remaining cells were fixed in 4% paraformaldehyde for 30 min and stained with crystal violet (0.2%). The number of invasion cells was counted.

### 2.9 Tube formation assay

Each well of the 24-well plate was coated with 100 µL of matrigel and incubated at 37°C for 30 min. Cells were then laid with 10,000 cells per well, cultured for 4 h, and observed under a microscope.

### 2.10 Statistics

GraphPad 7.0 was used for performing statistical analysis. Data were represented as mean ± SEM. Student’s *t*-test was used for statistical comparisons, and *p* < 0.05 was considered significant.

## 3 Results

### 3.1 SOX4 was upregulated in DB model mice, and its depletion rescued the viability of HRCECs caused by high glucose

**Figure 1:**
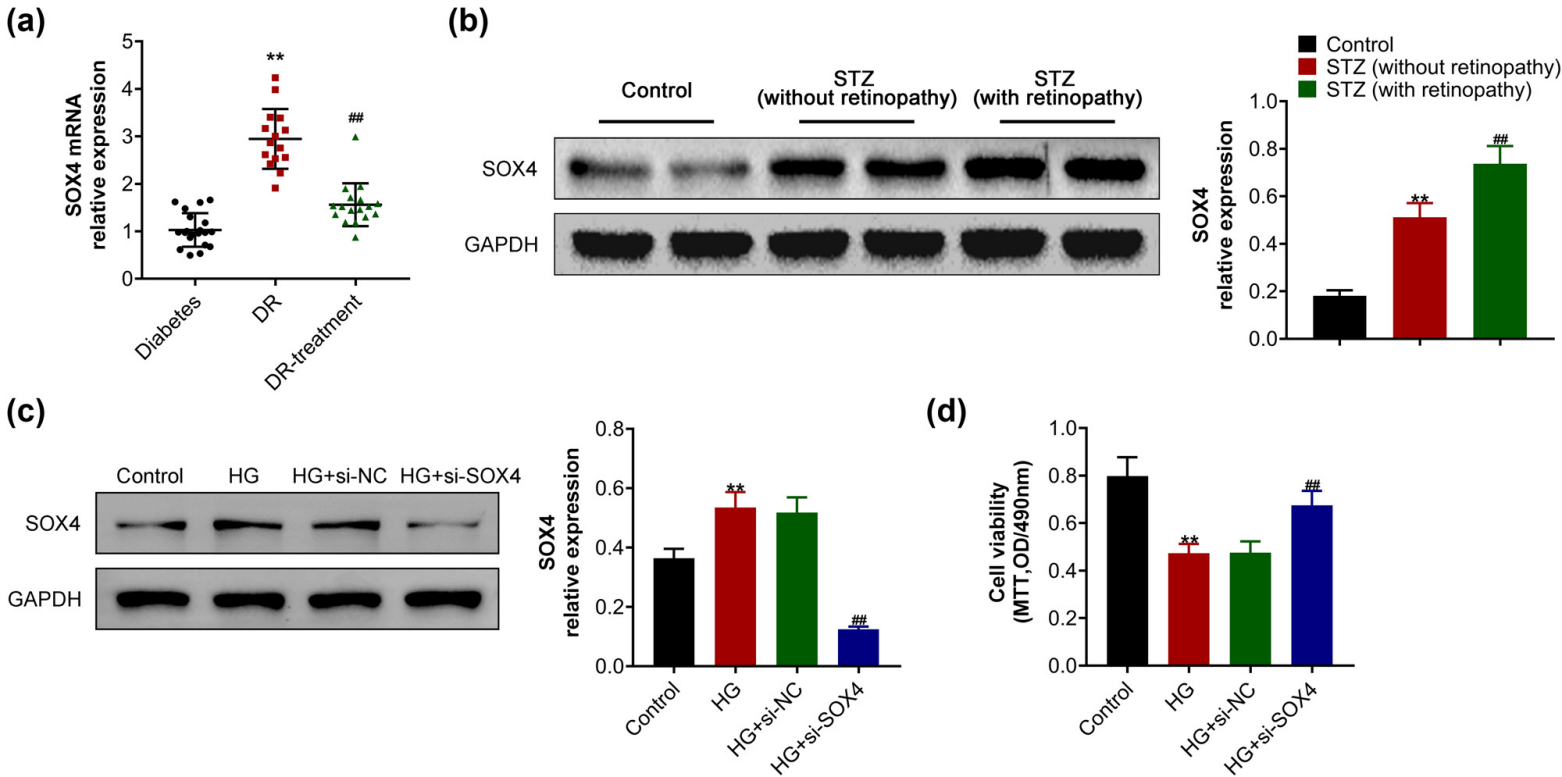
SOX4 was upregulated in DB samples, and its depletion suppressed the viability of high-glucose-induced HRCECs. (a) qPCR assays showed the mRNA level of SOX4 in the peripheral blood monocytes from patients with diabetes, DR, and DR-treatment group. (b) Immunoblot assay showed the expression of SOX4 in mice upon treatment of control, STZ (without retinopathy), and STZ (with retinopathy). (c) Immunoblot assay showed the expression of SOX4 in HRCECs upon the indicated treatment. (d) MTT assay showed the viability of HRCECs upon the indicated treatment. Results are presented as mean ± SEM, HG versus control, **p < 0.01, HG + siSOX4 versus HG + siNC, ^##^ *p* < 0.01. HG, high glucose, NC, negative control.

To uncover the possible effects of SOX4 on the progression of DR, the mRNA level of SOX4 was detected in the peripheral blood monocytes of patients. qPCR assays showed an increased SOX4 mRNA level in patients with DR, whereas it was decreased in the DR-treatment group (Figure 1a). The diabetic mice model was induced by STZ, and the expression of SOX4 increased, while it was aggravated in STZ-induced mice with retinopathy (Figure 1b). A DR cell model was constructed by administering high glucose (33 mM) into HRCECs for 24 h. Immunoblot assays showed the upregulation of SOX4 in high-glucose-induced HRCECs, compared to the normal group (Figure 1c). Subsequently, a siRNA of SOX4 was transfected into high-glucose-induced HRCECs to deplete its expression, and obviously decreased SOX4 expression was noticed (Figure 1c). MTT assay showed that high glucose treatment suppressed the viability of HRCECs, and SOX4 depletion increased the viability of HRCECs upon high glucose treatment (Figure 1d). Therefore, SOX4 was upregulated in DB samples, and its depletion rescued the viability of HRCECs caused by high glucose treatment.

### 3.2 SOX4 depletion restrained the high-glucose-induced inflammatory response in HRCECs

**Figure 2:**
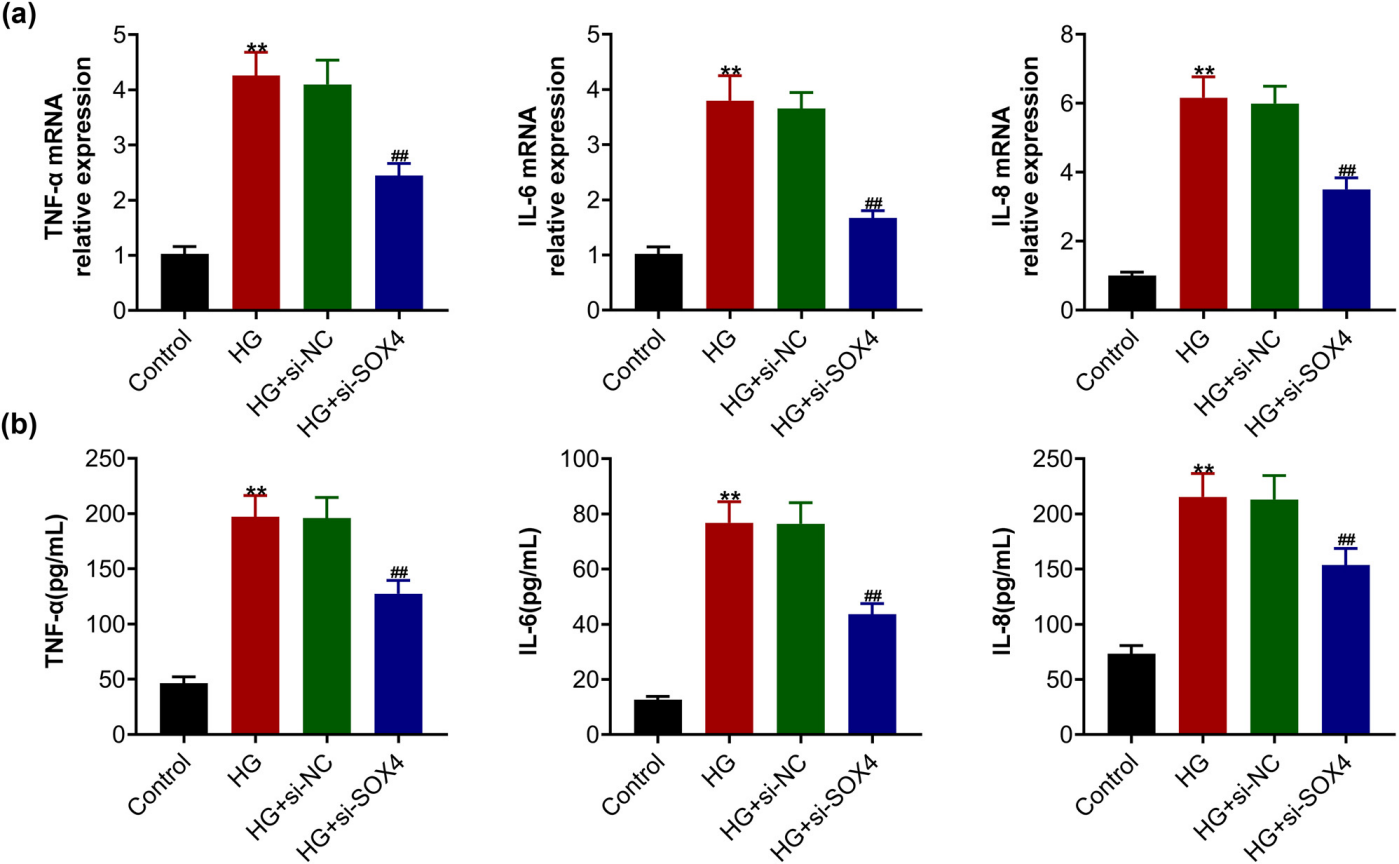
SOX4 depletion restrained the high-glucose-induced inflammatory response in HRCECs. (a) qPCR assay showed the mRNA levels of TNF-α, IL-6, and IL-8 in HRCECs upon the indicated treatment. (b) ELISA assay showed the secretion of TNF-α, IL-6, and IL-8 in HRCECs upon the indicated treatment. Results are presented as mean ± SEM, HG versus control, **p < 0.01, HG + siSOX4 versus HG + siNC, ^##^ *p* < 0.01. HG, high glucose, NC, negative control.

The following studies were then performed to detect the effects of SOX4 on the inflammatory response of HRCECs. qPCR assays showed that high glucose treatment increased the mRNA levels of inflammatory factors, including TNF-α, IL-6, and IL-8 in HRCECs (Figure 2a). In addition, SOX4 depletion suppressed the mRNA levels of these inflammatory factors, suggesting the inhibition of inflammatory response caused by SOX4 depletion (Figure 2a). Similarly, ELISA showed that high glucose treatment increased the secretion of TNF-α, IL-6, and IL-8 in HRCECs (Figure 2b), whereas SOX4 ablation suppressed the secretion of these inflammatory factors (Figure 2b). Therefore, SOX4 depletion restrained the high-glucose-induced inflammatory response in HRCECs.

### 3.3 SOX4 depletion suppressed the high-glucose-induced migration and angiogenesis in HRCECs

**Figure 3:**
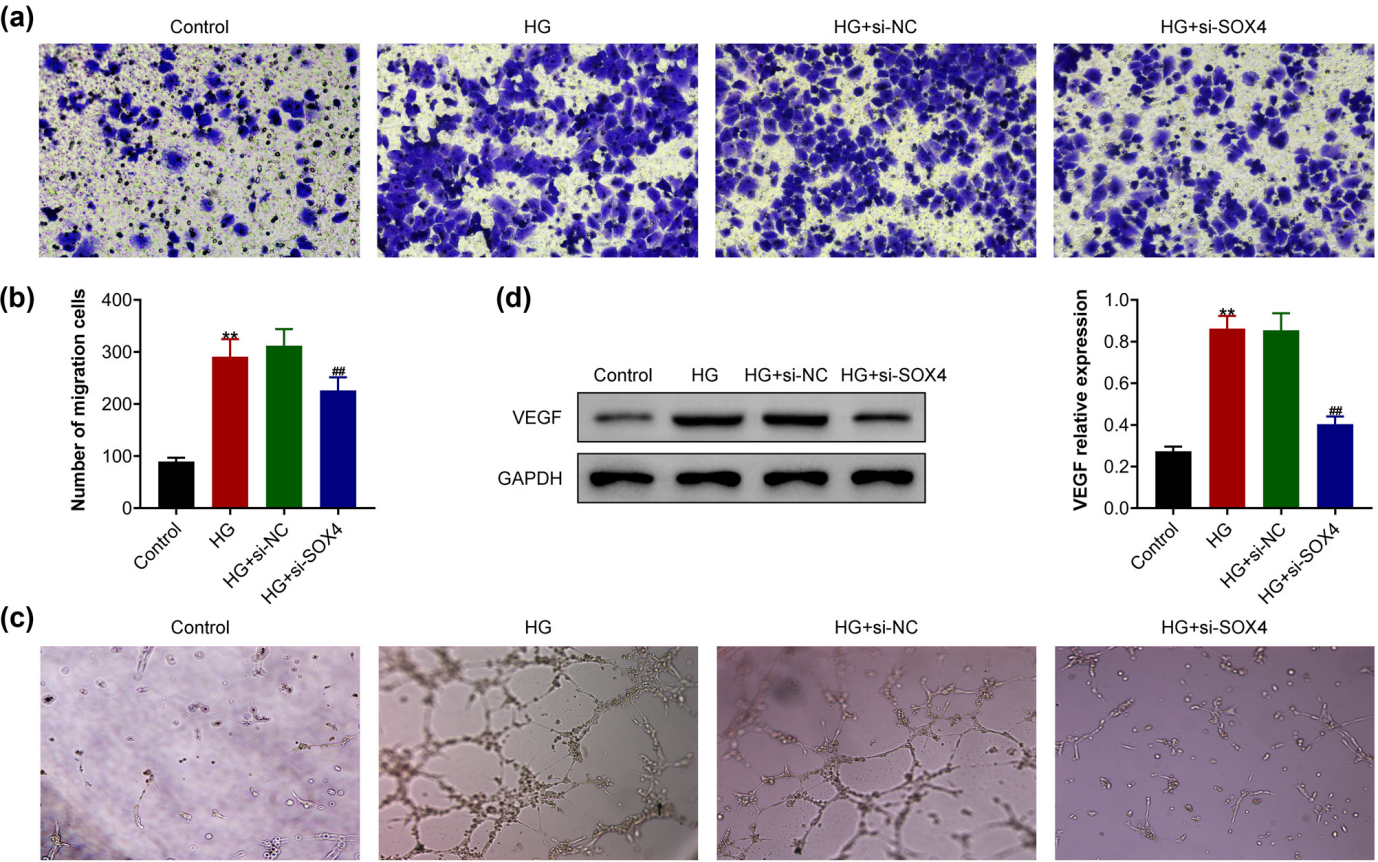
SOX4 depletion suppressed the high-glucose-induced migration and angiogenesis of HRCECs. (a) Transwell assay showed the migration of HRCEC upon the indicated treatment, and (b) the migratory cell numbers were counted. (c) Tube formation assay showed the angiogenesis of HRCECs upon the indicated treatment. (d) Immunoblot assay showed VEGF expression in HRCECs upon the indicated treatment. Results are presented as mean ± SEM, HG versus control, **p < 0.01, HG + siSOX4 versus HG + siNC, ^##^ *p* < 0.01. HG, high glucose, NC, negative control.

Subsequently, the present study detected the effects of SOX4 on the migration and angiogenesis of HRCECs upon high glucose treatment. Transwell assay showed that high glucose treatment promoted the migration of HRCECs, whereas the ablation of SOX4 suppressed the migration of HRCECs upon high glucose treatment (Figures 3a and b). The tube formation *in vitro* assay showed that high glucose treatment stimulated the tube formation of HRCECs, whereas the depletion of SOX4 suppressed the angiogenesis of HRCECs induced by high glucose (Figure 3c). In addition, the immunoblot assay showed that high glucose increased the expression of VEGF, whereas knockdown of SOX4 suppressed VEGF expression in HRCECs caused by high glucose (Figure 3d). Taken together, SOX4 depletion suppressed the high-glucose-induced migration and angiogenesis in HRCECs.

### 3.4 SOX4 ablation inhibited the high-glucose-induced NF-κB signaling pathway in HRCECs

**Figure 4:**
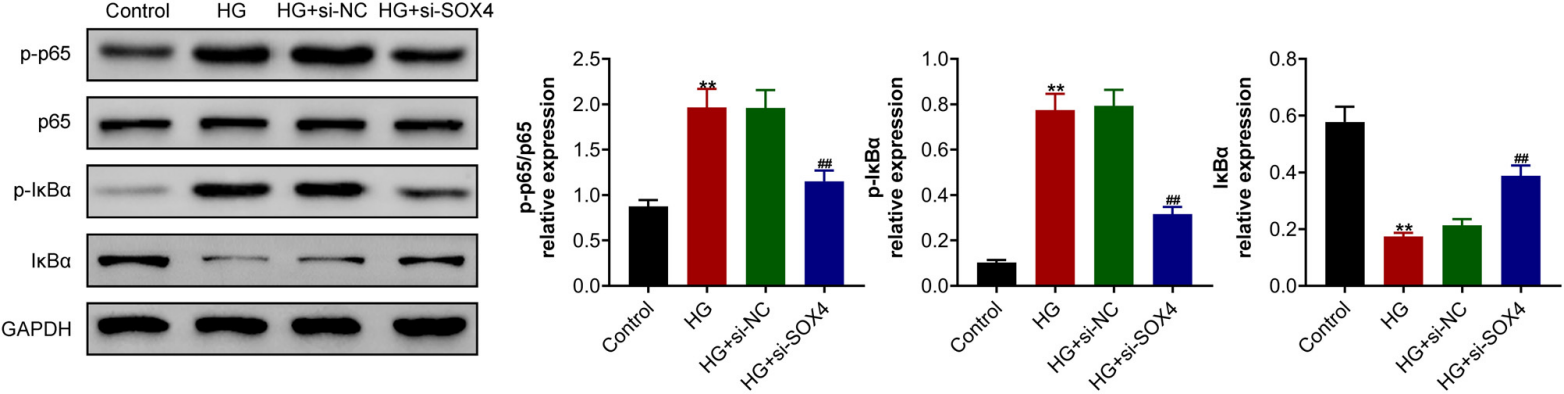
SOX4 ablation inhibited the high-glucose-induced NF-κB signaling pathway in HRCECs. Immunoblot assay showed the expressions of the phosphorylation levels of p65 and IκBα in HRCECs upon the indicated treatment. Results are presented as mean ± SEM, HG versus control, **p < 0.01, HG + siSOX4 versus HG + siNC, ^##^ *p* < 0.01. HG, high glucose, NC, negative control.

Further studies were performed to explore the possible mechanism. A previous study showed the effects of SOX4 on the NF-κB signaling pathway, which could affect cell viability, inflammation, and angiogenesis [19]. Therefore, the effects of SOX4 on the NF-κB signaling pathway were explored in high-glucose-induced HRCECs. Immunoblot assay showed that high glucose treatment increased the phosphorylation levels of p65 and IκBα in HRCECs (Figure 4). Importantly, depletion of SOX4 suppressed the phosphorylation levels of p65 and IκBα in HRCECs induced by high glucose (Figure 4). Taken together, SOX4 ablation inhibited NF-κB signaling pathway in HRCECs induced by high glucose.

## 4 Discussion

DR is the most important manifestation of diabetic microangiopathy, a kind of fundus lesion with specific changes, and is one of the serious complications of diabetes [20]. Vitrectomy should be performed as early as possible in patients with severe proliferative DR, such as repeated massive fundus hemorrhage, large fibrous proliferative membrane, and retinal detachment [21]. To further improve the cure rate of DR, new and more effective treatment drugs still need to be developed. Also, it is urgently needed to clarify the pathogenesis of DR and identify the key regulatory proteins [22]. Interestingly, this study identified a protein, SOX4, that could promote high-glucose-induced inflammation and angiogenesis of retinal endothelial cells, serving as a promising target for DR.

MTT assay showed the stimulative effects of SOX4 on the viability of high-glucose-induced HRCECs. qPCR and ELISA assays further confirmed the regulatory effects of SOX4 on the inflammatory response of high-glucose-induced HRCECs. Transwell, tube formation, and immunoblot assays were further performed and confirmed the regulatory effects of SOX4 on the migration and angiogenesis of high glucose-induced HRCECs. SOX4, well-known as a transcription factor, was expressed in the pancreas and is essential for normal endocrine pancreatic development [23]. Also, SOX4 was involved in regulating multiple cellular processes, such as tumorigenesis [10]. SOX4-induced ARHGAP9 overexpression also promoted the progression of acute myeloid leukemia [24]. Azilsartan could prevent AGE-induced inflammatory response in human chondrocytes through inhibition of SOX4 [25]. Our data, therefore, showed the potential of SOX4 as a target for DR treatment.

A previous study indicated that SOX4 could promote melanoma cell migration and invasion by activating the NF-κB pathway [19]. In this study, SOX4 was involved in the high-glucose-induced injury of HRCECs by regulating the NF-κB pathway. The pathway affected multiple downstream cellular processes, such as cell proliferation, motility, apoptosis, and angiogenesis, thus contributing to the progression of DR [17]. Besides, multiple proteins or drugs affected the progression and development of DR via the NF-κB pathway [2]. For example, nimbolide could ameliorate the STZ-induced DR in rats through the inhibition of the NF-κB pathway [26]. In addition, asiatic acid attenuated DR through NF-κB p65-mediated modulation of microglia polarization [27]. CD146 pathway was a novel biomarker of angiogenesis and inflammation in DR progression [27]. Similarly, SOX4 affected angiogenesis and inflammation in high-glucose-induced HRCECs.

Notably, overexpression of SOX4-mediated CXCL12 promoter in HCC cells enhanced tumor-induced angiogenesis, contributing to distant tumor metastasis and poor prognosis in hepatocellular carcinoma (HCC) patients [11]. In this study, the effects of SOX4 on the angiogenesis of HRCECs were determined. However, the precise mechanism underlying the effects of SOX4 on angiogenesis via the NF-κB pathway needs further study.

In conclusion, SOX4 depletion increased the viability, inhibited the inflammation level, and suppressed the migration and angiogenesis in high-glucose-induced HRCECs. The underlying mechanism was via the NF-κB pathway. SOX4 was therefore served as a promising target for DR treatment.
